# Flipping a coin in your head without monitoring outcomes? Comments on predicting free choices and a demo program

**DOI:** 10.3389/fpsyg.2013.00925

**Published:** 2013-12-09

**Authors:** Martin Lages, Stephanie C. Boyle, Katarzyna Jaworska

**Affiliations:** School of Psychology, University of GlasgowGlasgow, UK

**Keywords:** free will controversy, sequential dependencies, multivariate pattern analysis, free choice, prediction, searchlight, frontopolar cortex

In a recent study Soon et al. (2013) predicted abstract intentions from fMRI BOLD activities in localized areas of the brain. Activities in a spherical cluster of voxels served as input to a multivariate pattern classifier (linear SVM). The accuracy for predicting the intention to add or subtract two numbers was determined for clusters centered on different voxels. A prediction accuracy of 60% averaged across participants and based on 10-fold cross-validation was achieved for patterns of voxel activities in the *medial frontopolar cortex* and *precuneus* up to 4 s *before* participants reported being consciously aware of their decision. The prediction accuracy in this study was similar to studies on predicting spontaneous left or right motor decisions (Soon et al., 2008; Bode et al., 2011). Since the task demands placed on the participants create similar methodological issues as in previous studies (Lages and Jaworska, 2012), it seems possible that the multivariate classifier picked up sequential information processing between trials (Bode et al., 2012).

Although the average prediction accuracy of 60% returned to chance level for patterns of voxel activity in the two brain areas shortly after the onset of a new trial and remained at 50% between trials, this observation is neither necessary nor sufficient for the *absence* of sequential information processing. In order to investigate sequential dependencies the outcome of at least one preceding trial and the current trial needs to be taken into account. Depending on task and response, sequential information processing between trials may emerge in distributed form within the default mode network (DMN) at variable time points (Guggisberg and Mottaz, 2013). In the following we illustrate the issue and suggest how the data may be analyzed.

To illustrate sequential effects consider the following inconspicuous sequence of ten responses (S, A, A, A, S, A, S, A, S, S) where A and S stand for freely choosing addition and subtraction, respectively. There are five As and five Ss suggesting a binomial process with rate parameter *p* = 0.5. However, if we consider the nine subsequent pairs of responses {(S,A), (A,A), …, (S,S)} then we obtain unequal transition probabilities. A trained classifier that predicts the next response from the preceding response would be 3 out of 5 times or 60% correct if the preceding response is A and 3 out of 4 times or 75% correct if the response is S. Starting with a random guess in the first trial, this gives an average prediction accuracy of 65%. In two behavioral studies replicating two different choice tasks (Haynes et al., 2007; Soon et al., 2008) we used subsequent response pairs to train a linear classifier (SVM) and obtained an average prediction accuracy of 61.6 and 64.1%, respectively (Lages and Jaworska, 2012).

When asked to generate a random sequence, people typically alternate between binary responses with a probability of about 0.6 (Lopes, 1982). This response pattern appears to be relevant in Soon et al.'s study (2013) since the only behavioral evidence for memory-less choice in the 17 (out of 34) selected participants is a histogram plotting average frequencies for different lengths of response sequences fitted by an exponential distribution (Figure S1). The authors take the excellent fit as evidence for random performance. Recently Allefeld et al. (2013) released a detailed account of the behavioral data but there are no further details how the data were compiled and fit. Nevertheless, it is discernable from their Figure S1 that the fit represents an *exponential function* with two parameters rather than an *exponential distribution* with a single parameter and that observed frequencies do not add up to probability 1.0. In addition, an exponential distribution would only provide a meaningful approximation of the geometrically distributed phase lengths if choosing addition and subtraction were equally probable [*p* = (1−p) = 0.5]. However, even with a best-fitting rate parameter of 1-exp(−0.826) ≈0.56 the exponential distribution underestimates the relative frequency of alternations (A,S) and (S,A) with phase length 1 as well as repetitions (A,A,S) and (S,S,A) with phase length 2 (see Figure 1). Increased frequencies for short phase lengths are a hallmark of non-random human choice behavior (Wagenaar, 1972; Lopes, 1982; Treisman and Faulkner, 1987; Falk and Konold, 1997) and these characteristics are not only present in the behavioral data of Soon et al. (2013) but also in Soon et al. (2008); Bode et al. (2011), and Haynes et al. (2007) suggesting that free or spontaneous choice tasks result in non-random behavior.

**Figure 1:**
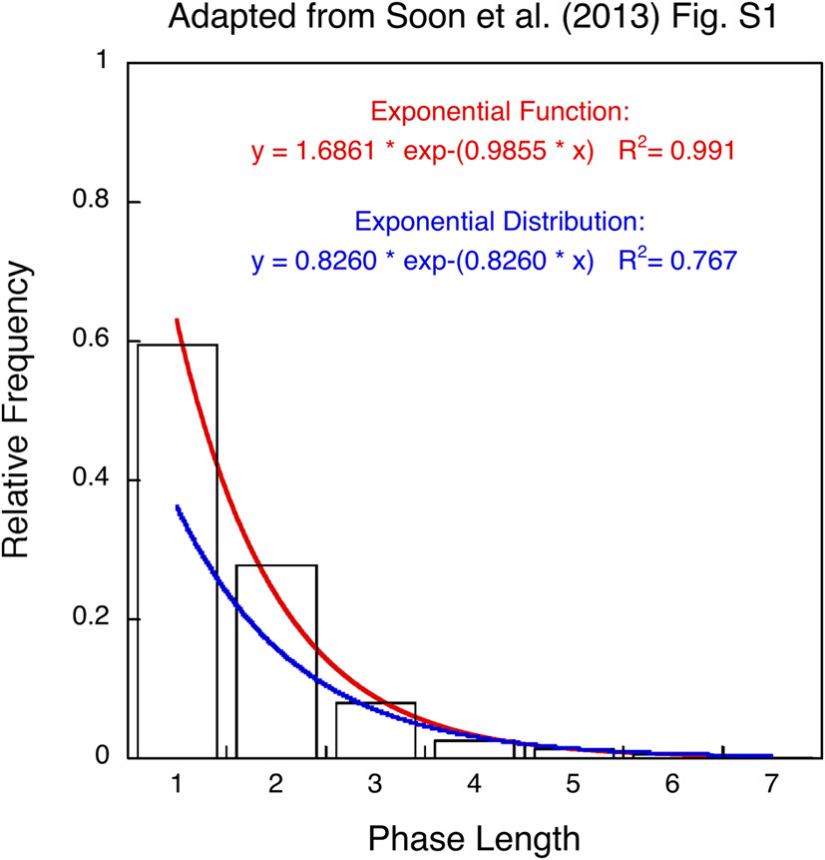
**Histogram for length of response sequences (phase length or runs) re-plotted as relative frequencies (adapted from Figure S1 in Soon et al., 2013).** The data points are fitted by an exponential function with two parameters (red curve) and an exponential distribution with a single parameter (blue curve). The red curve provides an excellent fit (*R*^2^ = 0.99) whereas the blue curve underestimates phase length 1 and 2 (*R*^2^ = 0.77). See text for explanation.

A related concern arises from the searchlight analyses. If patterns of voxel activities are analyzed within a moving spherical cluster to predict behavioral responses then pre-processing of the data and definition of the searchlight are important (Etzel et al., 2013; Todd et al., 2013). The implementation of regions of interest, temporal constraints (hemodynamic delay), pre-processing (covariates), and data selection can invalidate the results of a searchlight analysis (Kriegeskorte et al., 2009; Lindquist et al., 2009). In Soon et al. (2013) trials were selected (undersampled) in order to balance the mean response rate. It is therefore possible that the searchlight found a cluster of voxels that was predictive of the next response in the context of the preceding response, simply because transitions between successive responses remained unbalanced. A repeated choice task with self-monitoring of the decision process invites sequential dependencies because the observer has to remember goals, constraints, and execution of the task. If, for example, participants shift a decision criterion following each response (Lages and Treisman, 1998, 2010; Lages and Paul, 2006; Treisman and Lages, 2010) or engage in metacognition by recalling the last response before making the next then neural correlates of these response-dependent processes introduce a confound that would be picked up by a searchlight analysis as soon as transitions between response categories are unbalanced.

We recommend that rather than postulating a 50% chance level, prediction accuracy should be tested with a permutation test (Stelzer et al., 2013) and/or separate multivariate classification analyses conditional on the previous response. Only if individual prediction accuracies reliably exceed observable benchmarks such as response bias and transition probabilities can we rest assured that results are not confounded. The interested reader is invited to test predictability of their own free choice behavior by downloading the demo program in the Appendix.

## Appendix

*After the game is before the game—*Josef “Sepp” Herberger (1897-1977, football player and manager)

The demo program can be downloaded from http://www.psy.gla.ac.uk/~martinl/Assets/Software/Predictatron.txt

Please save the text file as Predictatron.m before running it under MatLab (MathWorks Inc., Natick MA). The program illustrates the difficulty for a human decision maker to generate a truly random sequence in a free choice task. Different choice tasks may be simulated by freely choosing between addition/subtraction (Haynes et al., 2007; Soon et al., 2013), left/right (Soon et al., 2008; Bode et al., 2011), or delay/non-delay (Filevich et al., 2013) before pressing the corresponding left or right arrow key on the keyboard. The program records 100 binary responses before it computes the rate of left/right key presses, number of left/right alternations, and left/right repetitions. Then the program determines the prediction accuracy of a linear support vector machine (SVM) using one preceding response to predict the next response (1-back SVM) for 10-fold cross-validation. If the algorithm does not exceed 60% prediction accuracy then two preceding responses are used to predict the next response (2-back SVM). If both prediction accuracies stay below 60% then the participant is considered “unpredictable” and wins against the “Predict-a-tron”. Results of the non-parametric Wald-Wolfowitz runstest() and parametric Lilliefors lillietest() as implemented in MatLab are also reported.
